# Novel Features Related to Polymers and the Environment

**DOI:** 10.3390/polym17121611

**Published:** 2025-06-10

**Authors:** Jesús-María García-Martínez, Emilia P. Collar

**Affiliations:** Polymer Engineering Group (GIP), Polymer Science and Technology Institute (ICTP), Spanish National Research Council (CSIC), C/Juan de la Cierva, 3, 28006 Madrid, Spain

In the early 1980s, the first global environmental crisis took place, focusing on the role of plastics in the substantial solid waste streams of major cities. It was evident then (and now) that the best environmental management practices required solid scientific and technical knowledge, often based on technical standards. Once at the end of their useful life, these plastics become involved in their materials (polymers and additives) in a circular economy strategy amidst the non-steady scenarios of the other key sectors of the economy, industry, society, and policy. Thus, forty years later, a twofold perspective (applied and academic) to link tandem polymers and the environment has led to a wide polymer research field devoted to continuously improving the environmental performance of polymers and polymer-based materials. This commitment to continuous improvement is a key aspect of the strategy. It comprises all the steps in the polymer management chain, from the raw materials to the polymers themselves, many of which come from classical and/or renewable sources (so-called bioplastics). There is a need to improve processability, ultimate properties, and performance through friendly environment additives, the recyclability of the materials, and innovative processes that will allow for better mechanical and/or energy recovery, including chemical recycling. These innovative processes that are at the forefront of polymer research and development hold promise for a more sustainable future, and should inspire optimism and hope [1,2,3,4].

Nevertheless, it is important to note that there is a great difference between prejudices and reality regarding classic plastics, and that these are still essential for the future [5]. Replacing such plastics in the market causes paradoxical negative consequences, since all that surrounds the actual living scenario is full of tradition-based polymeric materials with no counterparts in bio-based polymers. So, it makes no sense to demonize fuel-based polymers, but instead to help in their better circularity, since most current applications in medicine, energy, packaging, engineering plastics, and so on are based on these kinds of polymer-based materials [5,6]. Consequently, the scientific community must make a double effort to enhance R&D activities. That means the better management of polymeric residues and the realistic use of bio-based polymers when applicable [1,2,3,4,5,6,7]. Thus, jointly improving recycling routes in terms of scientific and technological development and economic considerations is a paramount concern for development in our sustainability-driven society [7], all without forgetting that polymer-based materials products are ubiquitous and necessary in our lives. Consequently, new advances in the corresponding technologies are needed to solve this task.

Therefore, this Special Issue includes many exciting works related to this frontrunner polymer R&D area [1,2,3,4]. The articles compiled in this volume fully match all the philosophical approaches mentioned above. Let us say that of the thirteen manuscripts submitted to this Special Issue, only seven were published (nearly 50% acceptance ratio) after the rigorous revision processes of *Polymers*. So, this issue has made a strenuous selection of the submitted articles to include just those in the field of Polymers and the Environment that are really at the forefront of research, avoiding including articles “reinventing the wheel” or that are not highly accurate (and so rejecting non-robust supported research) for such a controversial research field. Each article compiled in this volume fully matches the topic’s fundamentals, underscoring their significance. Since this editorial aims not to elaborate on each text but to encourage the reader to browse them in depth, these contributions have been briefly described below. For such purposes, a short note on each one has been reported to awaken interest in each of the contributions to this exciting Special Issue of *Polymers*, rather than providing an exhaustive description.

Hernández-Fernández et al. [8] propose a theoretical and experimental investigation regarding the inhibition of Ziegler–Natta catalyst on the synthesis of an ethylene-propylene copolymer based on the effect of traces of formaldehyde, propionaldehyde, and butyraldehyde. For such purposes, the authors analyzed 30 samples with different concentrations of these aldehydes and three control samples. The authors concluded that small amounts of formaldehyde (26 ppm), propionaldehyde (65.2 ppm), and butyraldehyde (181.2 ppm) considerably affect the productivity levels of the ZN catalyst, thus affecting the properties of the final product. Additionally, the computational analysis showed that the complexes formed by formaldehyde, propionaldehyde, and butyraldehyde with the active center of the catalyst are more stable than those obtained using the ethylene-Ti and propylene-Ti complexes.

Jamoussi et al. [9] utilized an experimental central statistical design to investigate the synthesis of a novel molecularly imprinted polymer. This polymer was designed for the selective extraction and enrichment of phenyl glyoxylic acid (PGA) in urine samples prior to high-performance liquid chromatography. The authors meticulously evaluated the structural and morphological characteristics of the molecularly imprinted and non-imprinted polymers using Fourier transform infrared spectroscopy (FT-IR) and scanning electron microscopy (SEM). They then optimized the molecularly imprinted solid-phase extraction. Under the optimized conditions, the molecularly imprinted polymers demonstrated an exceptional degree of selectivity and affinity for PGA, surpassing the results of previous studies. The recoveries of PGA were almost complete, ranging from 97.32% to 99.06%. Furthermore, the authors demonstrated the potential for recycling the polymers up to three times without a significant loss in analyte recovery, a testament to the impressive performance of their work.

In another exciting article, the team led by Weththimundi and Licchelli [10] examined the varnish formulations used by the master violin makers of the Italian Golden Age, particularly Antonio Stradivari, which combined siccative oils, such as linseed oil, with natural resins, including colophony. While these traditional varnishes are still applied to modern instruments, they can deteriorate over time due to mechanical stress, environmental conditions, and player contact. To enhance resistance against aging, the researchers recreated the historical varnish recipe and modified it with the cross-linking agent GLYMO (3-Glycidyloxypropyltrimethoxysilane). Both plain and functionalized varnishes underwent artificial aging tests, including exposure to UV light, temperature, and humidity variations. The results obtained indicate that the functionalized varnish exhibits improved durability, greater photostability, and enhanced scratch resistance, demonstrating its potential for preserving valuable string instruments.

With the ultimate purpose of recycling particle foams derived from expanded polypropylene, Schneider et al. [11] presented a new approach for utilizing a solid and dielectric heatable coating in the production of three-dimensional welded components from expanded polypropylene (ePP). It is well known that due to its chemical structure and resulting dielectric properties, the processing of the commonly used particle foam material, ePP, is limited. The latter is the reason why the authors analyzed three different types of water-soluble polymer polyvinyl alcohols (PVAs) as potential coating materials, adjusting the thermal and dielectric properties of the coating by adding glycerol, which influences both the temperature development in the radio-frequency (RF) welding process as well as the adhesive bond between the ePP foam particles. The authors found that this process is reversible if treated with hot water, which opens a new route for recycling particle foams.

The work by Silva et al. [12] has the objective of observing the wear presented by three polymeric materials, polyvinyl chloride (PVC), high-density polyethylene (HDPE), and polypropylene (PP) when exposed to chlorine in swimming pools and drinking water treatment plants. The authors based their study on chlorine’s ability to kill bacteria and fungi through a chemical reaction, suggesting that chlorine solutions are essential for cleaning and disinfecting public facilities. Consequently, the interest in studying its effect when in contact with different materials emerged. The authors conducted a thorough and well-designed study using accelerated degradation tests and various analytical methods (optical microscope, scanning electron microscope, and tensile tests), concluding that PVC exhibits more stable behavior under this scenario.

In their investigation, Fernandes et al. [13] explored cork composites derived from wine stopper production as durable and versatile materials used in industries such as the automotive, construction, and aerospace industries. It is well known that traditional agglomerated cork relies on a petrochemical-based polyurethane matrix. Instead, this research focuses on developing eco-friendly biocomposites using polyurethane modified with polyol derived from used cooking oil and cork. The authors evaluated the impact resistance of the samples, complementing the study with a finite element numerical model for compression simulation and validation. The results show that petrochemical polyol composites with a 3% addition of natural or modified cork achieved the highest specific absorbed energy. This finding not only demonstrates the feasibility of replacing petrochemical components with sustainable vegetable oil-based alternatives in polyurethane synthesis, but also provides practical insights for the application of these biocomposites in various industries.

Finally, and to demonstrate that interfacial agents obtained from polymer wastes are highly efficient in heterogeneous materials based on polymers, Collar and García-Martinez [14] established that this type of interfacial agent acts appropriately in compatibilizing the immiscible blend even in the most unfavorable possible scenario for compatibilization, as it is the inversion phase of a binary polymer blend. For such purposes, the authors examined the 50/50 polypropylene/polyamide 6 (iPP/PA6) system molded under confined flow conditions before and after modification with two different interfacial agents. The study employed Dynamic Mechanical Analysis (DMA) to conclude the efficiency of such interfacial agents. Thus, as mentioned before, the authors employed two distinct interfacial modifiers: one with terminal, side, and bridge SA grafts (aPP-SASA), and another containing succinyl-fluoresceine (SF) with bridge succinic anhydride grafting atactic polypropylene (aPP-SFSA). Additionally, the results from WAXS and SAXS synchrotron tests concluded that there were neither changes in the crystal morphology of the iPP or PA6 in the blends nor any co-crystallization process throughout the compositional range explored. In addition, a rough approximation predicting the interfacial effect of the interfacial agents is underlined by the molecular simulation of the interaction between the interfacial agent and the amino groups of the PA6 phase, supporting the conclusions obtained from the dynamic mechanical measurements.

Finally, and as a concluding remark, it is important to note that any topic related to the interaction of polymer systems and the environment is an essential and fascinating research activity at the forefront of the field of Polymer Science and Technology. This is a field that is constantly evolving and gaining interest in both academia and industrial scenarios, and will continue to do so in the near future. This is why we are excited to announce a new Special Issue on a related topic named “Polymers for Environmental Applications”, to be published in 2025 in *Polymers*. We are now open for submissions and we eagerly await your fine contributions to this dynamic and growing field.
